# MicroRNA-30 inhibits antiapoptotic factor Mcl-1 in mouse and human hematopoietic cells after radiation exposure

**DOI:** 10.1007/s10495-016-1238-1

**Published:** 2016-03-31

**Authors:** Xiang Hong Li, Cam T. Ha, Mang Xiao

**Affiliations:** Radiation Countermeasures Program, Armed Forces Radiobiology Research Institute, Uniformed Services University of the Health Sciences, Bethesda, MD USA

**Keywords:** Mcl-1, MiR-30, Radiation-injury, Hematopoietic cells, Apoptosis, Mice, Human CD34+ cells

## Abstract

We previously reported that microRNA-30 (miR-30) expression was initiated by radiation-induced proinflammatory factor IL-1β and NFkB activation in mouse and human hematopoietic cells. However, the downstream effectors of miR-30 and its specific role in radiation-induced cell death are not well understood. In the present study, we evaluated effects of radiation on miR-30 expression and activation of intrinsic apoptotic pathway Bcl-2 family factors in in vivo mouse and in vitro human hematopoietic cells. CD2F1 mice and human CD34+ cells were exposed to different doses of gamma-radiation. In addition to survival studies, mouse blood, bone marrow (BM) and spleen cells and human CD34+ cells were collected at 4 h, and 1, 3 and 4 days after irradiation to determine apoptotic and stress response signals. Our results showed that mouse serum miR-30, DNA damage marker γ-H2AX in BM, and Bim, Bax and Bak expression, cytochrome c release, and caspase-3 and -7 activation in BM and/or spleen cells were upregulated in a radiation dose-dependent manner. Antiapoptotic factor Mcl-1 was significantly downregulated, whereas Bcl-2 was less changed or unaltered in the irradiated mouse cells and human CD34+ cells. Furthermore, a putative miR-30 binding site was found in the 3′ UTR of Mcl-1 mRNA. miR-30 directly inhibits the expression of Mcl-1 through binding to its target sequence, which was demonstrated by a luciferase reporter assay, and the finding that Mcl-1 was uninhibited by irradiation in miR-30 knockdown CD34+ cells. Bcl-2 expression was not affected by miR-30. Our data suggest miR-30 plays a key role in radiation-induced apoptosis through directly targeting Mcl-1in hematopoietic cells.

## Introduction

More than 50 % of cancer patients receive radiotherapy, which often results in side effects due to radiation damage on normal tissue [1]. In addition, nuclear proliferation, terrorist activities, and the distribution of radioactive materials increase the risk of incidents involving radiation injuries. The hematopoietic system is one of the most radiation-sensitive organs [2]. Ionizing radiation doses above 1 Gy in humans pose a risk of BM failure [3].

Exposure to γ-radiation causes damage to DNA, protein, and lipids in mammalian cells, leading to “danger signals” and antigen release. These events could impact cellular tissue homeostasis and subsequent cell cycle checkpoint arrest, apoptosis, and stress-related responses. The effects of radiation on mitochondrial outer membrane permeabilization (MOMP) are required for activation of the caspase proteases that cause apoptotic cell death [4]. Radiation as an intrinsic apoptotic stimulus initiates mammalian cells’ apoptotic program by inhibiting Bcl-2 family antiapoptosis factor Bcl-2, Mcl-1 and Bcl-x, and triggering conformational changes in Bcl-2-associate X (Bax) and Bcl-2-antagonist/killer (Bak) proteins that enable oligomer formation on the mitochondria, causing MOMP and cytochrome c and other apoptogenic proteins in the intermembrane space to leak out [5]. Overexpression of Bcl-2 increases hematopoietic stem cell number and repopulation potential in lethally irradiated mice [6].

MicroRNAs (miRNA) are a class of small noncoding RNA molecules (on average 22 nucleotides long) found in eukaryotic cells. They have the ability to post-transcriptionally regulate gene expression via targeting the 3′ untranslated region (UTR) of messenger RNA transcripts (mRNAs) [7, 8]. miRNA-mediated gene repression occurs through both translational repression and mRNA destabilization [9]. Mammalian genomes encode thousands of conserved miRNAs that target mammalian genes and are abundant in many cell types [10, 11]. miRNAs could regulate the cellular changes required to establish stress-induced cell damage phenotypes [12]. On the other hand, miRNA also can be regulated during its maturation process, from primary and precursor to mature miRNA [13], although the underlying mechanisms are not well understood.

We previously reported that radiation upregulated miR-30b and miR-30c in human hematopoietic CD34+ cells, and miR-30 played a key role in radiation-induced human hematopoietic and their niche osteoblast cell damage through negatively regulating expression of survival factor REDD1 (regulated in development and DNA damage responses 1) and inducing apoptosis in these cells. Knockdown of miR-30c before radiation significantly increased clonogenicity in irradiated CD34+ cells [14]. Recently, we further reported that miR-30 expression in mouse BM, liver, jejunum and serum was initiated by radiation-induced proinflammatory factor IL-1β and NFkB activation. Delta-tocotrienol (DT3), a radioprotector, suppressed miR-30 and protected mice and human CD34+ cells from radiation exposure [15]. Our results from both in vitro and in vivo studies suggested miR-30 is an apoptosis inducer after radiation exposure. However the specific role of miR-30 in radiation-induced apoptotic cell death and its downstream target factors which caused mouse and human hematopoietic cell damage are not well understood. In this study, we extend our findings using human hematopoietic stem and progenitor CD34+ cells and an in vivo mouse model, to explore the effects and mechanisms of miR-30 on regulation of apoptotic cell death signaling in hematopoietic cells after γ-radiation.

## Materials and methods

### Mice and animal care

Twelve- to 14-week-old CD2F1 male mice (Harlan Laboratories, Indianapolis, IN) were used according to methods described in previous reports [16]. All animals were housed in an AAALAC-approved facility at the Armed Forces Radiobiology Research Institute (AFRRI), and the animal study protocol was approved by the Institutional Animal Care and Use Committee (IACUC). Mice were acclimatized upon arrival and representative animals were screened for evidence of disease. Animal rooms were maintained at 20–26 °C with 30–70 % humidity on a 12 h light/dark cycle. Commercial rodent chow (Harlan Teklad Rodent Diet 8604) was available ad libitum as was acidified water (pH 2.5–3.0) to control opportunistic infections. Animals were chosen randomly for each experimental group.

### Human CD34+ cells

Human primary hematopoietic CD34+ cells were provided by the Fred Hutchinson Cancer Research Center (Seattle, WA) as described in previous reports [14]. Thawed CD34+ cells were cultured in serum-free medium consisting of Iscove’s Modified Dulbecco’s Medium (IMDM) supplemented with BIT 9500 (Stem Cell Technologies, Tukwila, WA) and penicillin/streptomycin. Recombinant human (rh) stem cell factor (SCF, 100 ng/ml), rh flt-3 ligand (FL, 100 ng/ml) and rh interleukin-3 (IL-3, 25 ng/ml) were added. All cytokines were purchased from PeproTech, Inc. (Rocky Hill, NJ). The cells were incubated at 37 °C with 5 % CO_2_ [17].

### Irradiation procedure

Mice received whole-body irradiation (WBI) in a bilateral radiation field at AFRRI’s ^60^Co facility. The alanine/electron spin resonance (ESR) dosimetry system (American Society for Testing and Materials, Standard E 1607) was used to measure dose rates (to water) in the cores of acrylic mouse phantoms. Control animals were sham-irradiated, treated in the same manner as the irradiated animals, except that the ^60^Co source was not raised from the shielding water pool. The midline tissue doses to the mice were 5, 8 or 9 Gy at a dose rate of 0.6 Gy/min [16]. Survival was monitored for 30 days after WBI (N = 20/group). Separate groups of mice were used for other assays at each of the four time points (4 h, 1, 3, and 4 days) after sham or γ-irradiation (N = 6/time point). After irradiation, mice were returned to their home cages with food and water provided as usual. The day of irradiation was considered day 0.

Human CD34+ cells were irradiated at doses of 0, 0.5, 1.0 or 2.0 Gy (0.6 Gy/min); 1.0 and 2.0 Gy are lethal radiation doses of CD34+ cells and 2.0 Gy had been previously determined to generate one log of cell kill by clonogenic assay [17]. After irradiation, cells were washed once and cultured in fresh culture medium.

### Mouse peripheral blood cell counts and serum and tissue preparation

At 4 h and 1, 3, 4 and 7 days after WBI, mice were humanely euthanized for whole blood, serum and tissue collection as described in previous reports [18]. The mice were deeply anesthetized prior to collecting whole blood through a cardiac blood draw and confirmatory cervical dislocation was performed while the animal was still anesthetized in accordance with the approved IACUC protocol. The blood was immediately divided into two tubes. The samples in EDTA tubes were used for peripheral blood cell counts by a clinical hematology analyzer (Bayer Advia 120, Bayer, Tarrytown, NY) at the AFRRI Veterinary Sciences Department facility, and samples in BD Microtainer Gold tubes were left unmoved on racks. Following 30 min coagulation at room temperature, sera were well separated from the gel by 10 min-centrifugation at 10,000×*g*, collected and stored at −80 °C for later study. After blood collection and euthanasia were completed for an individual mouse, tissues were collected. BM cells were collected from mouse femurs and humeri. After erythrocytes were lysed with lysis buffer (Qiagen GmbH, Hilden, Germany), total BM myeloid cells were collected for further use. Mouse spleens were excised, rinsed with phosphate buffered saline (PBS), and snap-frozen in liquid nitrogen, then stored at −80 °C for further use.

### Micro-RNA (miRNA) extraction and quantitative real-time polymerase chain reaction (PCR)

Total RNA and miRNA from human CD34+ cells were extracted using mirVana miRNA isolation kits (Life Technologies) and miRNA from mouse serum was isolated using mirVana PARIS Kit (Ambion, Cat#AM1556) following the manufacturer’s protocol as reported previously [14, 15]. RNA concentrations were determined by measuring OD on a NanoDrop spectrophotometer ND-1000 (Thermo Fisher Scientific, Waltham, MA) and total RNA quality was verified on the Agilent 2100 bioanalyzer (Agilent Technologies, Palo Alto, CA) with RNA 6000 Nano chips. Reverse transcription (RT) was performed using TaqMan^®^ MicroRNA Reverse Transcription Kits (Applied Biosystems, Foster City, CA) according to the manufacturer’s instructions, and the resulting cDNAs were quantitatively amplified in triplicate for miR-30b and -30c expression using TaqMan^®^ MicroRNA specific primers for miR30b (ID#000602), miR30c (ID#000419) and U6 (ID#001973) on an IQ5 Real-Time PCR System (Bio-Rad, Hercules, CA). miRNA levels were normalized to U6 as an internal control [14].

### Pre-miRNA, miRNA inhibitor transfection

Pre-miR30 (PM11060), miR30-inhibitor (AM11060) or control-miRNA were purchased from Thermo Fisher Scientific (Grand Island, NY) and transfected into CD34+ cells using the Lipofectamine RNAiMAX (Cat# 13778-075, Invitrogen) according to the manufacturer’s protocol discussed in our previous report [14]. In brief, the diluted small RNA was mixed with Lipofectamine RNAiMAX transfection agent and incubated at room temperature, and then dispensed into a culture plate. We then overlaid 10^6^ CD34+ cell suspensions onto the transfection complexes and gently tilted the plate to mix. Cells were incubated at 37 ^°^C for 24 h and then harvested for further analysis.

### Protein extraction and immunoblotting

The frozen mouse tissues or cultured human CD34+ cells were homogenized in 1 × radio-immunoprecipitation lysis buffer (RIPA, Sigma-Aldrich, St Louis, MO) supplemented with a protease inhibitor tablet by tissue homogenizer (Fast Prep-24, MP Biomedicals, Solon, OH), following manufacturer recommendations. After 15 min centrifugation at 12,000×*g*, the supernatant was collected and protein concentrations were determined using a BCA assay kit (Pierce, Rockford, IL). The collected cell homogenates were denatured in Laemmli buffer supplemented with DTT (dithiothreitol), and the same amount of protein from each sample (100–120 µg) was loaded for SDS-PAGE electrophoresis. Subsequently, immunoblotting was performed following standard procedures with an enhanced chemiluminescence kit (Thermo Scientific, Rockford, IL). The images were captured by CCD camera and the optical densities of protein bands were quantified using ImageGauge software as previously described [15]. Antibodies for Mcl-1, Bcl-X_L_, Bcl-2, Bim, Bax, Bak, γH2AX, cytochrome c, Caspase-3 and caspase-7 were purchased from Cell Signaling (Minneapolis, MN) and Santa Cruz (Santa Cruz Biotechnology, Dallas, TX), and beta-actin was obtained from Sigma-Aldrich (St Louis, MO).

### Clonogenic assay

Total live BM myeloid cells from each mouse and human CD34+ cells were measured by Trypan blue staining. Clonogenicity of mouse BM cells and human CD34+ cells was quantified in standard semisolid cultures in triplicate using 1 ml of Methocult GF + system for either mouse cells or human cells (StemCell Technologies) according to the manufacturer’s instructions, as described previously [15]. Briefly, mouse BM cells from pooled samples or CD34+ cells from liquid culture were washed twice with IMDM (Iscove’s Modified Dulbecco’s Media) and seeded at 1 × 10^4^ cells/dish (mouse cells) or 3 × 10^3^ cells/dish (CD34+ cells) in 35-cm cell culture dishes (BD Biosciences). Plates were scored for colonies after culturing for 10 days (for mouse colonies) or 14 days (for human colonies) at 37 °C in 5 % CO_2_.

### Luciferase reporter assay

To construct pMIR-REPORT-Mcl-1-3′ UTR-WT and pMIR-REPORT-Mcl-1-3′ UTR-MUT plasmids, a wild type 3′ UTR segment of human Mcl-1 mRNA (1198-1725 nt, Genebank accession No. NM_021960) containing two putative miR-30 binding sites (1329–1351 and 1584–1602 nt) or a corresponding multi-base mutant sequence was cloned into the SacI and HindIII sites downstream of the firefly luciferase reporter gene in pMIR-REPORT Luciferase (Ambion, Austin, TX, USA) by BioInnovatise, Inc. (Rockville, MD). These reporters were transfected into human CD34+ cells using Lipofectamine 2000 (Thermo Fisher Scientific, Grand Island, NY, USA) and transfection efficiency was corrected by a p-MIR-report-β-gal control vector (Ambion, Austin, TX, USA). The Ambion pre-miR-30 precursors were co-transfected with pMIR-report, pMIR-hMcl-1-WT, or pMIR-hMcl-1-MUT plasmid. Luciferase and β-galactosidase activity were determined using Dual-Light System (Applied Biosystems, Bedford, MA, USA).

### Statistical analysis

Fisher’s exact test was used to compare survival among groups at the end of 30 days. Differences between means were compared by ANOVA and Student’s *t* tests. p < 0.05 was considered statistically significant. Results are presented as means ± standard deviations or standard errors of the mean as indicated.

## Results

### 30-day survival study of mice exposed to ^60^Co-radiation

**Fig. 1:**
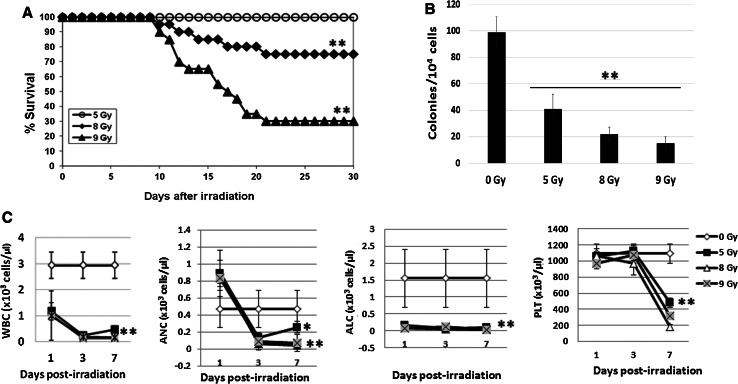
30-day survival study, BM cell clonogenicity and blood cell counts in mice after ^60^Co whole-body irradiation (WBI). **a** CD2F1 mice were irradiated with a single radiation dose of 5, 8 or 9 Gy, at a dose rate of 0.6 Gy/min in the AFRRI ^60^Co radiation facility (N = 20/group). The 30-day survival curves for 5, 8 and 9 Gy reflect an approximate LD0/30, LD25/30, and LD70/30, respectively. Mean ± SD. ** p < 0.01, 5 Gy irradiation vs. 8 or 9 Gy irradiated controls. **b** Clonogenicity of mouse BM cells was quantified in standard semisolid cultures in triplicate. Colonies were counted 10 days later. Results were from one representative experiment of two independent experiments (N = 6 mice/point/experiment). Mean ± SD. ** p < 0.01, radiation vs. sham-irradiated controls. **c** Total white blood cells (WBC), absolute neutrophil counts (ANC), absolute lymphocyte counts (ALC), and platelets (PLT) were measured in whole blood samples 1, 3 and 7 days after radiation (N = 6). Mean ± SD. * p < 0.05; ** p < 0.01, radiation vs. sham-irradiated controls

CD2F1 male mice were whole-body irradiated (WBI) with a single radiation dose 5, 8 or 9 Gy, at a dose rate of 0.6 Gy/min in the AFRRI ^60^Co radiation facility (N = 20/group). Figure 1a illustrates the 30-day survival curves for mice exposed to 5, 8, and 9 Gy; survival rates were as follows: 5 Gy (100 %), 8 Gy (75 %), and 9 Gy (30 %). Lethal doses of 8 or 9 Gy caused significant animal death compared with the 5 Gy sublethal radiation dose.

### Radiation inhibited mouse BM hematopoietic stem and progenitor and peripheral blood cells

BM cells were collected from femurs and humeri of mice 24 h after 5, 8 and 9 Gy irradiation. Total live BM myeloid cells from each mouse were measured by trypan blue staining. Clonogenicity was compared between samples collected from individual mice after different doses of WBI. Figure 1b shows the significant decreased colony numbers in irradiated mouse BM, in comparison with sham-irradiated control (N = 6, p < 0.01). Furthermore, peripheral blood was collected from sham- or γ-irradiated mice. Blood counts were measured on 1, 3 and 7 days post-irradiation. Consistent with clonogenicity results, a severe reduction in radiation-induced blood cells was observed in mice that received 5, 8 or 9 Gy of WBI. Figure 1c illustrates the total white blood cells (WBC), absolute neutrophil counts (ANC), absolute lymphocyte counts (ALC), and platelets (PLT) measured in whole blood at the indicated time points post-irradiation (N = 6). WBC and ALC were significantly reduced for all radiation doses at day 1 after irradiation and remained below baseline levels though the last time point, 7 days after irradiation. ANC gradually decreased from day 1 to day 3 for all radiation doses and began to recover by day 7 after 5 Gy irradiation, whereas mice exposed to radiation doses >5 Gy were exhibited low ANC levels through day 7. The loss of PLT started later and dropped sharply after day 3, in a radiation dose-dependent manner. Reductions in red blood cell counts were minor after WBI (data not shown).

### Radiation induced apoptotic factor activation in mouse BM and spleen cells

**Fig. 2:**
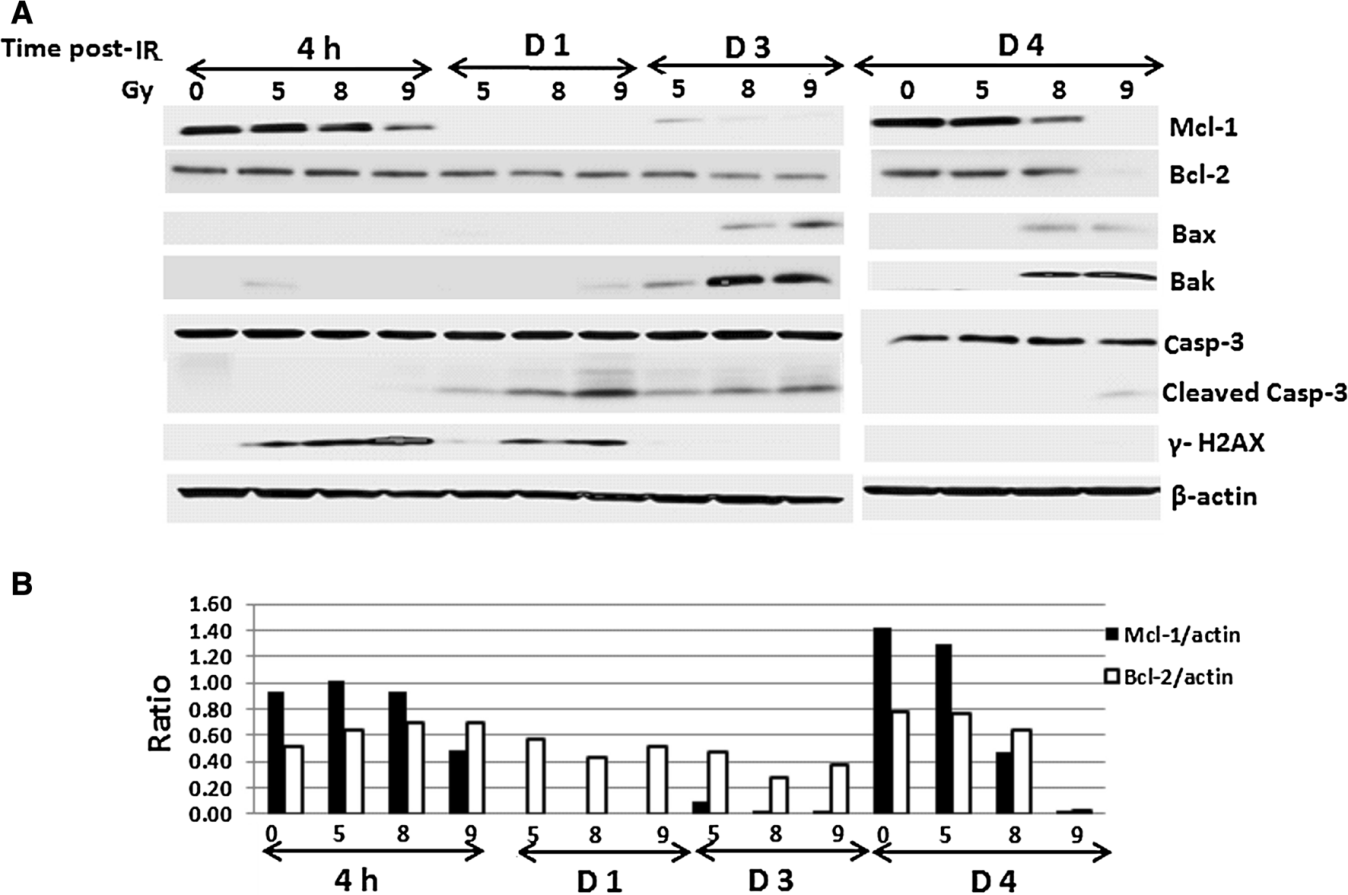
Radiation induced apoptotic factor activation in mouse BM cells. BM cells were collected from mouse femurs and humeri at 4 h, and 1, 3 and 4 days after 5, 8 or 9 Gy irradiation, and lysates were generated as pooled samples (N = 6). **a** Immunoblotting was performed to determine protein expression of Mcl-1, Bcl-2, Bax, Bak, γ-H2AX, and caspase-3 in these samples. **b** β-Actin was used as a control for sample loading. The ratios of Mcl-1 and Bcl-2 vs. β-actin are shown. Results are from one representative experiment of two independent experiments (6 mice/group/experiment)

It was suggested that Mcl-1 is essential for survival of early cells, including embryonic cells, and hematopoietic stem and progenitor cells [19]. In contrast, anti-apoptotic effects of Bcl-2 were observed in mature cells [20]. To identify impacts of radiation on apoptosis of hematopoietic stem and progenitor cells, we examined antiapoptotic factors Bcl-2, Bcl-XL and Mcl-1 and proapoptotic factors Bax and Bak, as well as caspase-3 activation and γ-H2AX expression in mouse BM. BM cells were collected from mouse femurs and humeri at indicated times after 5, 8 or 9 Gy irradiation, and lysates were generated as pooled samples due to low cell numbers after irradiation (N = 6). Western blot results in Fig. 2a indicate DNA damage marker γ-H2AX upregulation was initiated at 4 h after 5–9 Gy WBI and was continually expressed up to 1 day after 8 and 9 Gy irradiation. Inhibition of Mcl-1 expression started at 4 h after 9 Gy, and Mcl-1 was undetectable from 1 to 3 days after 5-9 Gy. Four days after irradiation, Mcl-1 recovered to normal levels after 5 Gy, and partially recovered after 8 Gy. No Mcl-1 was observed in 9 Gy irradiated samples 4 days after WBI. Interestingly, the anti-apoptotic factor Bcl-2 in mouse BM cells was not significantly downregulated by 5–9 Gy radiation, except for 4 days after 9 Gy. The ratios of Mcl-1 and Bcl-2 vs. β-actin are shown in Fig. 2b. In contrast to Mcl-1 downregulation, levels of proapoptotic factors Bax and Bak were enhanced by radiation and the apoptosis executor caspase-3 was activated 1 day after WBI. Bcl-X_L_ protein was undetectable in mouse BM cells (data not shown).

**Fig. 3:**
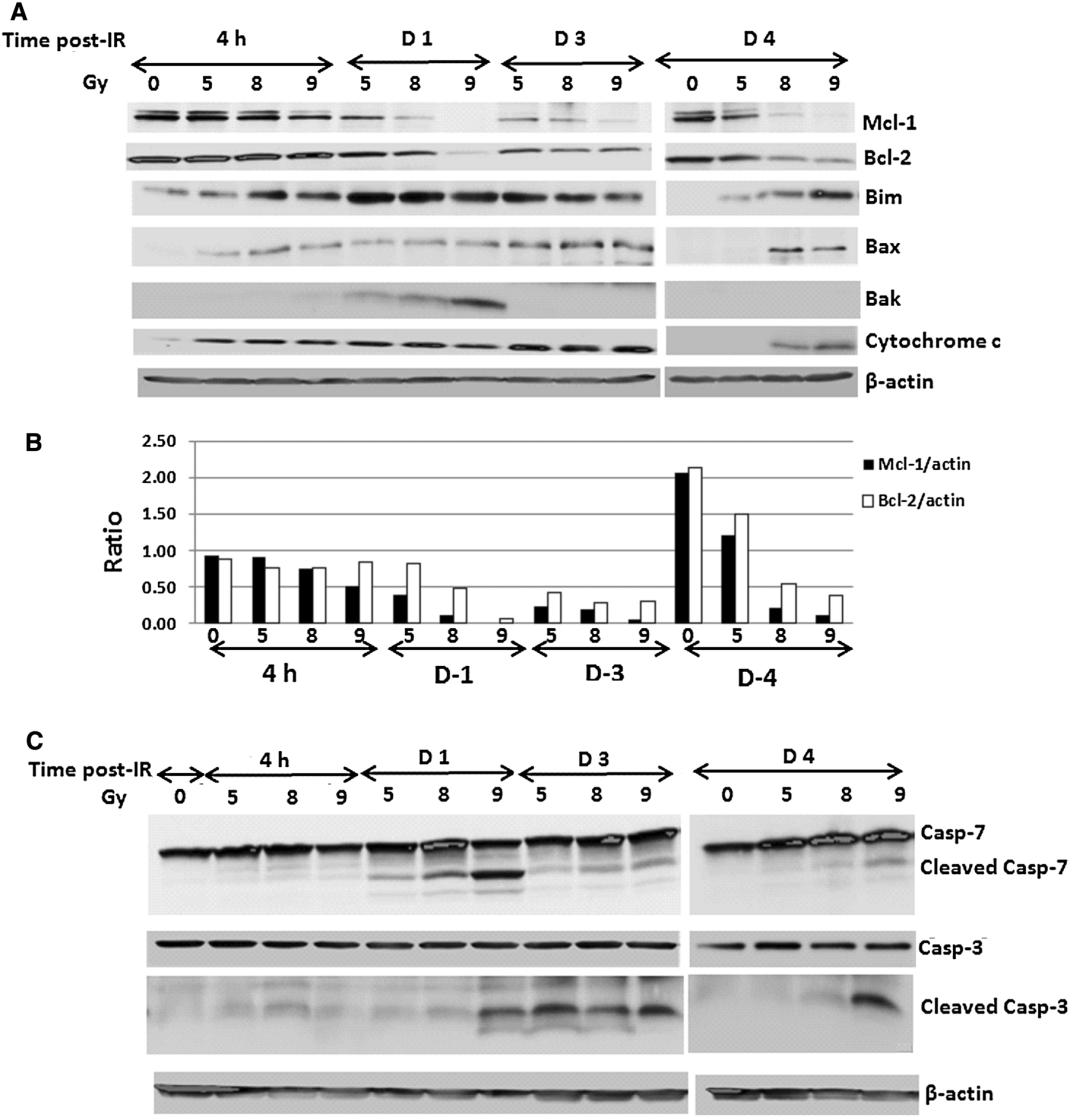
Radiation induced apoptotic factor activation in mouse spleen cells. Spleens were collected and whole spleen lysates from each group (N = 6) were generated as pooled samples. Immunoblotting was performed to determine protein expression of **a** Mcl-1, Bcl-2, Bim, Bax, Bak, and cytochrome c and **b** the ratios of Mcl-1 and Bcl-2 vs. β-actin. **c** Caspase-3 and caspase-7 expression and activation. β-actin was used as a control for sample loading. Results are from one of two experiments (6 mice/group/experiment)

We next examined radiation-induced apoptosis signaling in mouse spleen cells 4 h, and 1, 3 and 4 days after 0, 5, 8 or 9 Gy irradiation. Spleens were harvested (N = 6/group) and whole spleen lysates were generated as pooled samples. Immunoblotting for these samples was performed to analyze apoptotic cascade proteins, including antiapoptotic factors Mcl-1, Bcl-2, and Bcl-X_L_ and proapoptotic factors Bim, Bax and Bak. Radiation inhibited antiapoptotic factors and upregulated proapoptotic factors in a radiation dose-dependent manner as shown in Fig. 3a. Eight and 9 Gy WBI suppressed Mcl-1 expression at every time point; whereas, the decrease of Mcl-1 expression in 5 Gy irradiated spleen samples was observed 1 and 3 day post-WBI, and started recovery by day 4 after radiation. Effects of radiation on Bcl-2 expression were relatively minor; the ratios of Mcl-1 and Bcl-2 vs. β-actin are shown in Fig. 3b. The Bcl-X_L_ protein level was very low to undetectable in mouse spleen cells (data not shown). The irreversible apoptosis-inducer cytochrome c was highly expressed in all irradiated mouse spleen samples except those from 4 days after 5 Gy. Consistent with cytochrome c release, the activation of apoptotic executors caspase-3 and caspase-7, shown as cleaved lower molecular weight bands in the western blot image, occurred in the irradiated spleens and peaked at day 1(caspase-7) and day 3 (caspase-3) after WBI, respectively (Fig. 3c). Both caspase-3 and caspase-7 activation returned to undetectable baseline levels as in sham-irradiated samples 4 days after 5 Gy.

### Mcl-1 was highly suppressed by radiation in human CD34+ cells

**Fig. 4:**
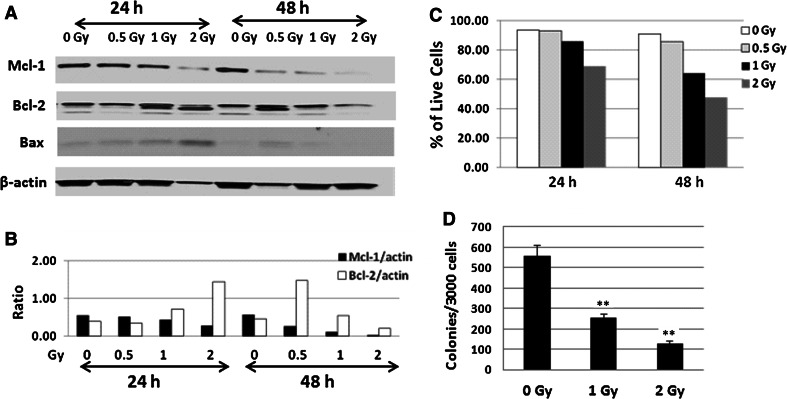
Mcl-1 was highly suppressed by radiation in human CD34+ cells. CD34+ cells were irradiated at doses of 0, 0.5, 1.0 or 2.0 Gy (0.6 Gy/min) and samples were collected at 24 and 48 h after irradiation. **a** Immunoblotting was performed to determine protein expression of Mcl-1, Bcl-2, and Bax in these samples. **b** β-Actin was used as a control for sample loading. The ratios of Mcl-1 and Bcl-2 vs. β-actin are shown. **c** Human CD34+ cell expansion and viability (trypan blue-negative cells) from all groups were quantified. **d** Clonogenicity of human hematopoietic progenitor CD34+ cells was quantified in standard semisolid cultures in triplicate. Colonies were counted 14 days later. Results from one representative experiment of three independent experiments were showed. Mean ± SD. ** p < 0.01, sham-control vs. irradiated

We next evaluated the effects of radiation on Mcl-1, Bcl-X_L_ and Bcl-2 expression in human CD34+ cells. The cells were irradiated at doses of 0, 0.5, 1.0 or 2.0 Gy and samples were collected at 24 and 48 h after irradiation. Data from the immunoblotting assay (Fig. 4a) showed Mcl-1 was suppressed, and Bax expression was highest, at 24 h after 2 Gy irradiation. At 48 h after 0.5–2 Gy of irradiation, Mcl-1 expression was further decreased. However, the impact of radiation on Bcl-2 expression in CD34+ cells was not significant as shown by Bcl-2/actin ratios (Fig. 4b). Bcl-X_L_ protein was undetectable in CD34+ cells (data not shown). Consistent with the Mcl-1 downregulation shown by immunoblotting, survival of CD34+ cells significantly decreased from 95 % (sham-irradiated) to 61 and 48 % 48 h after radiation at 1 and 2 Gy (Fig. 4c), and colony formation numbers decreased from 560 ± 30/3000 CD34+ cells (control) to 230 ± 15/3000 CD34+ cells (after 1 Gy) and 134 ± 11/3000 CD34+ cells (after 2 Gy) (Fig. 4d), (p < 0.01).

### Radiation-induced Mcl-1 downregulation was miRNA-30 dependent

**Fig. 5:**
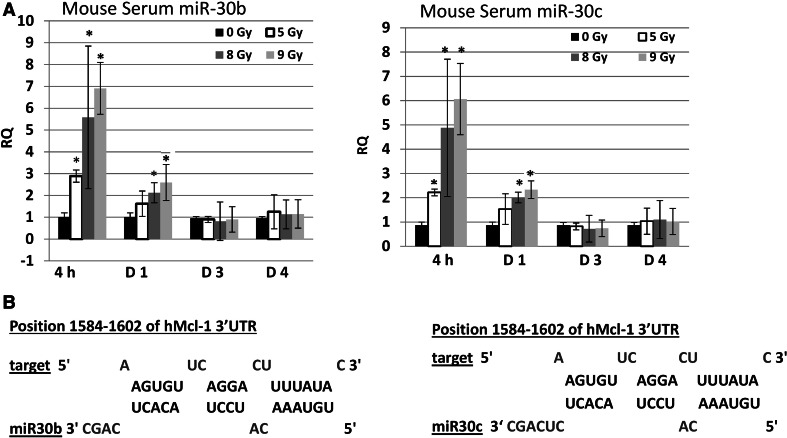
MiR-30 expression in mouse serum after γ-irradiation. MiRNAs were extracted from mouse serum. Levels of miR-30b and miR-30c expression were determined by Quantitative Real Time-RT PCR in mouse (**a**) Serum at 4 h, and 1, 3 and 4 days after 5, 8 or 9 Gy irradiation. U6 was used as a control. Results represent one of three independent experiments. *RQ* relative quantification. N = 6/group in each experiment; Mean ± SD. * p < 0.05, ** p < 0.01; radiation vs. sham-control. **b** MiR-30b and miR-30c binding sites in Mcl-1 3′UTR are shown

We previously reported that miR-30 played a key role in radiation-induced human CD34+ and osteoblast cell damage through an apoptotic pathway [14], and a radiation countermeasure candidate, delta-tocotrienol (DT3), suppressed radiation-induced miR-30 expression in mouse BM, liver, jejunum and serum, and in human CD34+ cells, and protected mouse and human CD34+ cells from radiation exposure [15]. In this study, expression of miR-30b and miR-30c was determined in mouse serum at 4 h, and 1, 3 and 4 days after 5, 8 or 9 Gy irradiation, since miR-30 levels in serum were parallel to expression in BM after radiation [15]. We found that both miR-30b and miR-30c were highly induced by 5–9 Gy within 4 h in serum, in a radiation dose-dependent manner (Fig. 5a). The levels of serum miR-30s 24 h post-radiation dropped in all samples, but were still significantly higher than control in 8 and 9 Gy irradiated samples as shown in Fig. 5a.

We further asked whether increases of miR-30 are responsible for radiation-induced Mcl-1 repression in hematopoietic cells. To answer this question, we analyzed potential targets of miR-30 family members using the miRNA target prediction database RNAhybrid 2.2 (http://bibiserv.techfak.uni-bielefeld.de/rnahybrid/) [21], and found that members of the miR-30 family were predicted to target the antiapoptosis factor Mcl-1. Figure 5b shows putative binding sites for miR-30b and miR-30c in the 3′UTR of the *Mcl*-*1* gene. Hence we explored interactions between the miR-30 family and Mcl-1.

**Fig. 6:**
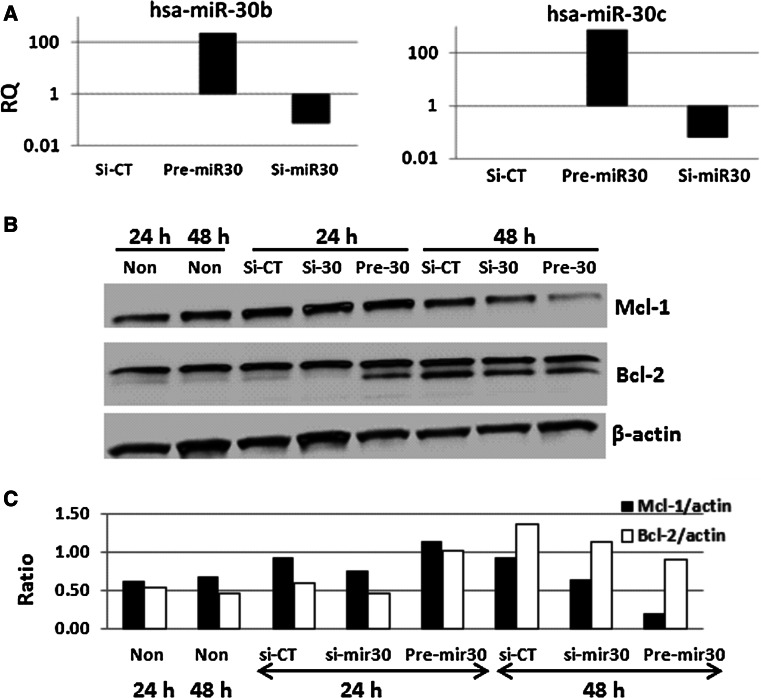
Over-expression of miR 30 in CD34+ cells inhibited Mcl-1 protein expression. Pre-miR30, miR30 inhibitor (si-miR30), or control miR (CT-miR) molecules were transfected into CD34+ cells. miR-30b and miR-30c expression were examined 24 h post-transfection by quantitative RT-PCR. U6 was used as a control. *RQ* relative quantification. **a** Transfection of pre-miR-30 enhanced both miR-30b and miR-30c expression more than 100-fold and transfection of inhibitors suppressed miR-30b and miR-30c expression by >50-fold in CD34+ cells. **b** Mcl-1 and Bcl-2 expression in non-transfected, miR-control, inhibitor and pre-miR-30 transfected CD34+ cells were evaluated by immunoblotting 24 and 48 h after transfection. Levels of Mcl-1 expression in CD34+ cells were significantly inhibited 48 h after pre-miR-30 transfection, whereas Bcl-2 was not impacted by miR-30 overexpression in these cells. **c** The ratios of Mcl-1 and Bcl-2 vs. β-actin were measured in different treatment groups

The effects of miR-30 on Mcl-1 expression in CD34+ cells were evaluated using gain and loss of miR-30 expression. CD34+ cells were transfected with miR-30 inhibitor, precursors (pre-miR30) or control-miR from Life Technologies Co.; miR-30b and miR-30c expression were examined by quantitative RT-PCR 24 h post-transfection and U6 was used as a control. Results shown in Fig. 6a demonstrate that transfection of pre-miR-30 enhanced both miR-30b and miR-30c expression more than 100-fold and transfection of inhibitor suppressed miR-30b and miR-30c expression by >50-fold in CD34+ cells. Western blot assays were used to test Mcl-1 and Bcl-2 expression in non-transfected, miR-control, inhibitor and pre-miR-30 transfected CD34+ cells as shown in Fig. 6b. Forty-eight h after pre-miR-30 transfection, the level of Mcl-1 expression in CD34+ cells was inhibited significantly, whereas no Mcl-1 downregulation was shown in control- or miR-30-inhibitor transfected samples compared with non-transfection control (Fig. 6c). Antiapoptosis factor Bcl-2 was not impacted by miR-30 overexpression in these cells (Fig. 6b, c).

### Knockdown of miR-30 blocked radiation-induced Mcl-1 reduction in CD34+ cells

**Fig. 7:**
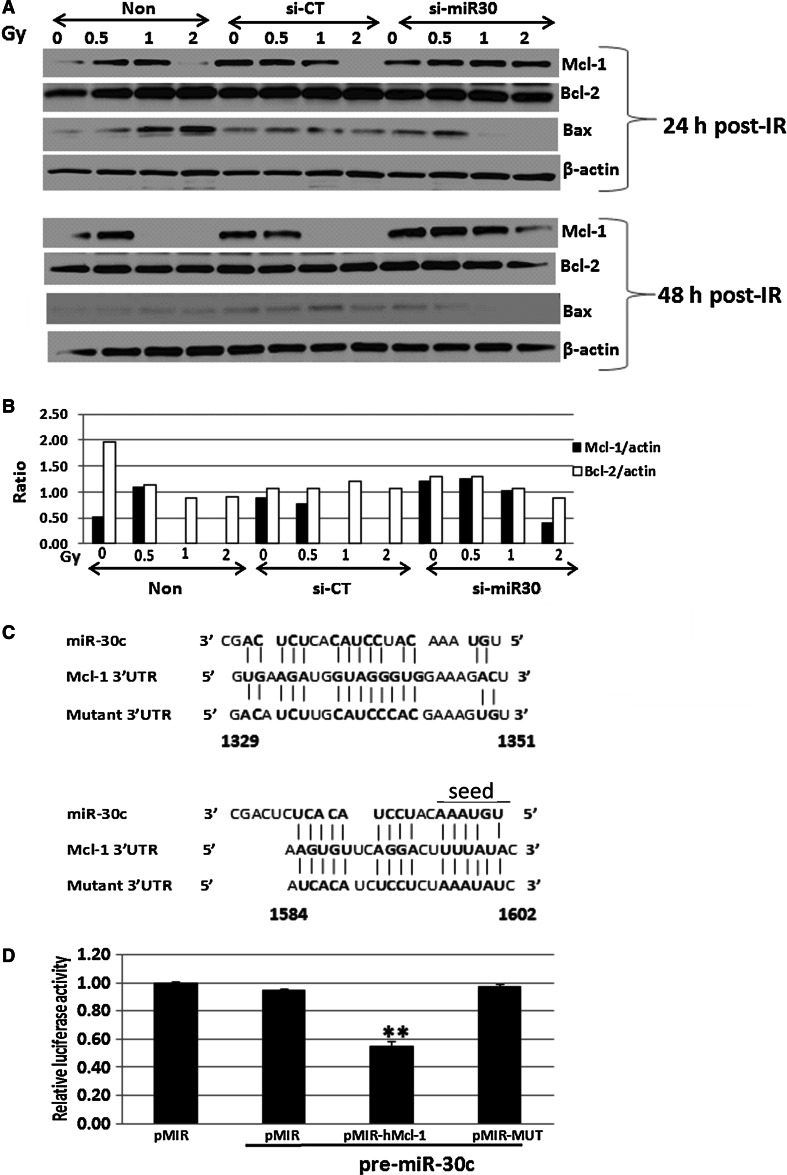
MiR-30 regulated Mcl-1 protein expression in CD34 + cells after γ-irradiation. **a** Endogenous Mcl-1, Bcl-2 and Bax protein levels were measured by western blot assays after non-, control-miR (CT-miR), or miR30-inhibitor transfection and γ-irradiation in CD34+ cells. Radiation downregulated Mcl-1 and enhanced Bax expression in non- or CT-miR transfected samples, whereas transfection of miR30-inhibitor maintained Mcl-1 protein levels and suppressed Bax expression in CD34+ cells 24 and 48 h after irradiation. Bcl-2 expression was not impacted by radiation and/or gene transfection. **b** The ratios of Mcl-1 and Bcl-2 vs. β-actin were measured in different treatment groups 48 h after 0, 0.5, 1, or 2 Gy irradiation. **c** Two putative miR-30 binding sites in the 3′UTR of Mcl-1 (1329–1351 and 1584–1602 nt) and the alignment of miR-30 with the 3′UTR insert are illustrated. A mutation was generated on the Mcl-1 3′-UTR sequence in the complementary site and the 5′end seed region of miR-30, as indicated. **d** The firefly luciferase p-MIR-report vector (pMIR) as a control, p-MIR-report vector with Mcl-1 3′UTR (pMIR-hMcl-1), and p-MIR-report vector with mutant 3′UTR (pMIR-MUT) were transiently transfected or cotransfected with an expression plasmid for pre-mir-30 into human CD34+ cells. Luciferase activity in CD34+ cells transfected with pMIR alone, or pre-miRNA-30 precursor cotransfected with pMIR-control, pMIR-hMcl-13′UTR, or pMIR-MUT 3′UTR is shown. Mean ± SD. (N = 3) ** p < 0.01; pMIR-hMcl-1 3′UTR cotransfected with pre-miRNA-30 compared to pMIR-control or pMIR-MUT 3′UTR and pre- miRNA-30 precursor cotransfected cells

We next examined the effects of miR-30 on Mcl-1 expression in CD34+ cells after radiation. The cells were exposed to different doses of γ-radiation at 24 h after non-transfection, miR-control, or miR-30 inhibitor transfection, and Mcl-1 and Bcl-2 protein expressions were tested by western blot in samples collected at 24 h (48 h post-transfection) and 48 h (72 h post- transfection) after irradiation. Figure 7a shows radiation upregulated proapoptotic factor Bax and downregulated Mcl-1 expression in non-transfected and control-miR-transfected CD34+ cells 24 h after 2 Gy and 48 h after 1 and 2 Gy irradiation, respectively. Transfection of miR-30 inhibitor significantly protected Mcl-1 from radiation-mediated downregulation and maintained the Mcl-1 levels as in sham-irradiated CD34+ cells. As expected, Bcl-2 expression was not changed by radiation nor miR-30 inhibition in CD34+ cells. The ratio of Mcl-1 or Bcl-2 vs. β-actin at 48 h post-irradiation is shown in Fig. 7b. In addition, radiation-induced Bax expression was completely blocked by knockdown of miR-30 in CD34+ cells.

Next, we asked whether the negative effect of miR-30 on Mcl-1 expression was direct. To answer this question, we set up a luciferase reporter assay as described in Materials and Methods. The putative miR-30 binding sites were predicted using target prediction programs RNAhybrid 2.2 [21]. There are two putative miR-30 binding sites in the 3′UTR of Mcl-1 (1329-1351 and 1584–1602 nt, with the 5′ end of the miR-30 seed sequence in the latter) and the alignment of miR-30 with the 3′UTR insert is illustrated in Fig. 7c. The firefly luciferase -report vector plasmid (p-MIR, Ambion, Austin, TX, USA) was modified by insertion of the Mcl-1-derived mir-30 binding sites or a multi-base mutant into the 3′UTR. The p-MIR-report vector with Mcl-1 3′UTR (pMIR-hMcl-1 3′UTR) and p-MIR-report vector with mutant 3′UTR (pMIR-MUT 3′UTR) were constructed by BioInnovatise, Inc. (Rockville, MD). These reporters were transfected into human CD34+ cells, and transfection efficiency was corrected by a p-MIR-report-β-gal control vector (Ambion, Austin, TX, USA). The Pre-miRNA-30 Precursor was co-transfected where indicated in Fig. 7d. As shown in Fig. 7d, cotransfection of CD34+ cells with the parental firefly luciferase reporter construct (pMIR-vector control) plus the pre-mir-30 does not significantly change the expression of the reporter. However, when the mir-30 target site from the Mcl-1 3′UTR is inserted into the luciferase construct (pMIR-hMcl-1), expression of luciferase is strongly decreased when cotransfected with pre-miR-30. This suppression is abolished by mutation of the binding sites (pMIR-MUT). The effect of miR-30 occurred only when both miR-30 and its target sequence were present; suggesting that miR-30 directly inhibits the expression of Mcl-1 through binding to its target sequence in Mcl-1gene.

## Discussion

Hematopoietic cells are very sensitive to radiation and ≥1 Gy of radiation could result in DNA damage and apoptosis in these cells. Our results in Fig. 1 confirm whole-body irradiation (WBI)-induces severe mouse hematopoietic cell damage, and even a 5 Gy sublethal dose of WBI caused critical decreases in hematopoietic stem and progenitor cells in mouse BM and suppressed mouse blood cell counts. It was suggested that radiation as an intrinsic apoptotic stimulus initiates mammalian cells’ apoptotic program [5]. However, the nature of the anti- and pro-apoptosis regulation in response to radiation is largely unknown. Understanding individual contributions of radiation-induced apoptosis is important in the development of new radioprotection, mitigation as well as treatment strategies. Our results demonstrate that antiapoptosis factor Mcl-1 is very sensitive to γ-radiation and moderate doses (5 Gy WBI in mouse and 0.5 Gy in human CD34+ cells) of radiation can suppress Mcl-1 expression in both mouse BM and spleen and human CD34+ cells. In contrast, Bcl-2 expression was relatively stable after radiation exposure in these cells. Bcl-X_L_ expression in irradiated and unirradiated mouse BM and spleen cells, and CD34+ cells, was very low to undetectable in our study.

Although Bcl-2, Bcl-X_L_ and Mcl-1 share Bcl-2 homology (BH) domains [22], differential effects of these antiapoptotic Bcl-2 family members on survival of cells have been reported [23–26]. Among them, Mcl-1 is structurally unique in comparison with other antiapoptotic Bcl-2 family members. Mcl-1 has a longer amino terminus (N-terminus) than other Bcl-2 family members, which contains numbers of functional modification regions [27, 28]. On the outer mitochondrial membrane (OMM), Mcl-1, similar to other antiapoptotic Bcl-2 family members, inhibits apoptotic cell death by binding and sequestering the proapoptotic proteins Bax and Bak. However, when targeted to the mitochondrial matrix (Matrix), Mcl-1 initiates mitochondrial function, including promoting normal ATP production, efficient oxidative phosphorylation, and reducing production of reactive oxygen species [28, 29]. These two separable functions may play critical roles in promoting cell survival. Thus, Mcl-1 may possess multiple functions on survival of cells.

It is well known that Mcl-1 is essential for embryonic development [19], survival of lymphocytes [30], hematopoietic stem cells [31] and neutrophils [32, 33]. Campbell et al. [31] reported that Mcl-1 but not Bcl-2 and Bcl-X_L_ was an indispensable regulator of self-renewal and is highly expressed in human hematopoietic stem cells. Knockdown of Mcl-1 in ontogenetically primitive human pluripotent stem cells resulted in complete ablation of stem cell self-renewal function. Further, *mcl*-*1* gene deletion caused severe reductions of mouse BM cells [34], whereas *Bcl*-*2* deficient mice did not show problems in early hematopoiesis [20]. Consistent with these reports, in our current study we found that Mcl-1 was significantly downregulated after radiation in mouse BM and human CD34+ cells, whereas Bcl-2 expression was relatively stable and not significantly impacted by radiation exposure in these cells.

Our previous studies suggested miR-30 is an apoptosis inducer in mouse and human hematopoietic cells. Radioprotector delta-tocotrienol suppressed miR-30 expression in mouse serum and cells and in human CD34+ cells, and protected mouse and human CD34+ cells from radiation exposure [14, 15]. However, the specific role of miR-30 in radiation-induced apoptotic cell death and its downstream target factors which caused mouse and human hematopoietic cells damage are not well understood. A number of studies have examined the general and specific effects of miRNA perturbation in radiation-exposed cells and evidence for miRNA involvement in the radiation response is increasing [35–37]. In the current study, we demonstrated increased levels of miR-30b and miR-30c in mouse serum after 5–9 Gy WBI, and this increase was radiation dose-dependent. Furthermore, we found putative miR-30 binding sites in the 3′UTR of Mcl-1 mRNA (Fig. 5b) and demonstrated for the first time that miR-30 directly inhibits the expression of Mcl-1 by binding to its target sequences (Fig. 7c, d). Previously we reported that knockdown of miR-30 before irradiation significantly increased clonogenicity in irradiated human CD34+ cells [14]. In the current study as shown in Fig. 7a and b, we further demonstrated that knockdown of miR-30 before irradiation in human CD34+ cells blocked radiation-induced reduction of Mcl-1, and the proapoptotic factor Bax was no longer increased by radiation. In contrast, Bcl-2 expression was not affected by miR-30 in these cells. Thus, our data from the current study suggest an important downstream target of miR-30 in irradiated hematopoietic cells is Mcl-1, and miR-30 is responsible for radiation-induced apoptosis in mouse and human hematopoietic cells through targeting the antiapoptotic factor Mcl-1.

## References

[1] Bentzen SM (2006). Preventing or reducing late side effects of radiation therapy: radiobiology meets molecular pathology. Nat Rev Cancer.

[2] Xiao M, Inal CE, Whitnall MH (2005). Effects of 5-androstenediol on survival, clonogenicity, and expression of IL-6 and NFkB in irradiated human osteoblast and hematopoietic CD34+ cells. Blood.

[3] Coleman CN, Stone HB, Moulder JE, Pellmar TC (2004). Medicine modulation of radiation injury. Science.

[4] Tait SW, Green DR (2010). Mitochondria and cell death: outer membrane permeabilization and beyond. Nat Rev Mol Cell Biol.

[5] Basanez G, Soane L, Hardwick JM (2012). A new view of the lethal apoptotic pore. PLoS Biol.

[6] Domen J, Cheshier SH, Weissman IL (2000). The role of apoptosis in the regulation of hematopoietic stem cells: overexpression of Bcl-2 increases both their number and repopulation potential. J Exp Med.

[7] Robertson B, Dalby AB, Karpilow J, Khvorova A, Leake D, Vermeulen A (2010). Specificity and functionality of microRNA inhibitors. Silence.

[8] Hu H, Gatti RA (2011). MicroRNAs: new players in the DNA damage response. J Mol Cell Biol.

[9] Djuranovic S, Nahvi A, Green R (2011). A parsimonious model for gene regulation by miRNAs. Science.

[10] Friedman RC, Farh KK, Burge CB, Bartel DP (2009). Most mammalian mRNAs are conserved targets of microRNAs. Genome Res.

[11] Wong N, Wang X (2015). miRDB: an online resource for microRNA target prediction and functional annotations. Nucleic Acids Res.

[12] Mendell JT, Olson EN (2012). MicroRNAs in stress signaling and human disease. Cell.

[13] Slezak-Prochazka I, Durmus S, Kroesen BJ, van den Berg A (2010). MicroRNAs, macrocontrol: regulation of miRNA processing. RNA.

[14] Li XH, Ha CT, Fu D, Xiao M (2012). Micro-RNA30c negatively regulates REDD1 expression in human hematopoietic and osteoblast cells after gamma-irradiation. PLoS ONE.

[15] Li XH, Ha CT, Fu D, Landauer MR, Ghosh SP, Xiao M (2015). Delta-tocotrienol suppresses radiation-induced microRNA-30 and protects mice and human CD34+ cells from radiation injury. PLoS ONE.

[16] Li XH, Ghosh SP, Ha CT, Fu D, Elliott TB, Bolduc DL, Villa V, Whitnall MH, Landauer MR, Xiao M (2013). Delta-tocotrienol protects mice from radiation-induced gastrointestinal injury. Radiat Res.

[17] Xiao M, Inal CE, Parekh VI, Chang CM, Whitnall MH (2007). 5-Androstenediol promotes survival of gamma-irradiated human hematopoietic progenitors through induction of nuclear factor-kappaB activation and granulocyte colony-stimulating factor expression. Mol Pharmacol.

[18] Li XH, Fu D, Latif NH, Mullaney CP, Ney PH, Mog SR, Whitnall MH, Srinivasan V, Xiao M (2010). Delta-tocotrienol protects mouse and human hematopoietic progenitors from gamma-irradiation through extracellular signal-regulated kinase/mammalian target of rapamycin signaling. Haematologica.

[19] Rinkenberger JL, Horning S, Klocke B, Roth K, Korsmeyer SJ (2000). Mcl-1 deficiency results in peri-implantation embryonic lethality. Genes Dev.

[20] Veis DJ, Sorenson CM, Shutter JR, Korsmeyer SJ (1993). Bcl-2-deficient mice demonstrate fulminant lymphoid apoptosis, polycystic kidneys, and hypopigmented hair. Cell.

[21] Rehmsmeier M, Steffen P, Hochsmann M, Giegerich R (2004). Fast and effective prediction of microRNA/target duplexes. RNA.

[22] Danial NN, Korsmeyer SJ (2004). Cell death: critical control points. Cell.

[23] Cory S (1995). Regulation of lymphocyte survival by the bcl-2 gene family. Annu Rev Immunol.

[24] Bodrug SE, Aime-Sempe C, Sato T, Krajewski S, Hanada M, Reed JC (1995). Biochemical and functional comparisons of Mcl-1 and Bcl-2 proteins: evidence for a novel mechanism of regulating Bcl-2 family protein function. Cell Death Differ.

[25] Ohta K, Iwai K, Kasahara Y, Taniguchi N, Krajewski S, Reed JC, Miyawaki T (1995). Immunoblot analysis of cellular expression of Bcl-2 family proteins, Bcl-2, Bax, Bcl-X and Mcl-1, in human peripheral blood and lymphoid tissues. Int Immunol.

[26] Rudner J, Elsaesser SJ, Muller AC, Belka C, Jendrossek V (2010). Differential effects of anti-apoptotic Bcl-2 family members Mcl-1, Bcl-2, and Bcl-xL on celecoxib-induced apoptosis. Biochem Pharmacol.

[27] Day CL, Chen L, Richardson SJ, Harrison PJ, Huang DC, Hinds MG (2005). Solution structure of prosurvival Mcl-1 and characterization of its binding by proapoptotic BH3-only ligands. J Biol Chem.

[28] Thomas LW, Lam C, Edwards SW (2010). Mcl-1; the molecular regulation of protein function. FEBS Lett.

[29] Perciavalle RM, Opferman JT (2013). Delving deeper: MCL-1′s contributions to normal and cancer biology. Trends Cell Biol.

[30] Tripathi P, Koss B, Opferman JT, Hildeman DA (2013). Mcl-1 antagonizes Bax/Bak to promote effector CD4(+) and CD8(+) T-cell responses. Cell Death Differ.

[31] Campbell CJ, Lee JB, Levadoux-Martin M, Wynder T, Xenocostas A, Leber B, Bhatia M (2010). The human stem cell hierarchy is defined by a functional dependence on Mcl-1 for self-renewal capacity. Blood.

[32] Milot E, Filep JG (2011). Regulation of neutrophil survival/apoptosis by Mcl-1. Scientific World J.

[33] Dzhagalov I, St John A, He YW (2007). The antiapoptotic protein Mcl-1 is essential for the survival of neutrophils but not macrophages. Blood.

[34] Opferman JT, Iwasaki H, Ong CC, Suh H, Mizuno S, Akashi K, Korsmeyer SJ (2005). Obligate role of anti-apoptotic MCL-1 in the survival of hematopoietic stem cells. Science.

[35] Metheetrairut C, Slack FJ (2013). MicroRNAs in the ionizing radiation response and in radiotherapy. Curr Opin Genet Dev.

[36] Chaudhry MA (2009). Real-time PCR analysis of micro-RNA expression in ionizing radiation-treated cells. Cancer Biother Radiopharm.

[37] Dickey JS, Zemp FJ, Martin OA, Kovalchuk O (2011). The role of miRNA in the direct and indirect effects of ionizing radiation. Radiat Environ Biophys.

